# Tethered primary hepatocyte spheroids on polystyrene multi-well plates for high-throughput drug safety testing

**DOI:** 10.1038/s41598-020-61699-4

**Published:** 2020-03-16

**Authors:** Farah Tasnim, Nisha Hari Singh, Elijah Keng Foo Tan, Jiangwa Xing, Huan Li, Sebastien Hissette, Sravanthy Manesh, Justina Fulwood, Kapish Gupta, Chan Way Ng, Shuoyu Xu, Jeffrey Hill, Hanry Yu

**Affiliations:** 1 Institute of Bioengineering and Nanotechnology, #04-01, 31 Biopolis Way, The Nanos, Singapore, 138669 Singapore; 2 Experimental Therapeutics Centre (ETC), Level 3, 31 Biopolis Way, The Nanos, Singapore, 138669 Singapore; 3 Mechanobiology Institute, T-Labs, #05-01, 5A Engineering Drive 1, Singapore, 117411 Singapore; 4 Yong Loo Lin School of Medicine (Department of Physiology) and Graduate School for Integrative Sciences & Engineering (NGS), National University of Singapore, MD9-04-11, 2 Medical Drive, Singapore, 117593 Singapore; 5 CAMP IRG, Singapore-MIT Alliance for Research and Technology, 1 CREATE Way, Enterprise Wing, Level 4, Singapore, 138602 Singapore; 6 Sussex Drug Discovery Centre, School of Life Sciences, University of Sussex, Brighton, BN19RH UK

**Keywords:** High-throughput screening, Biomaterials - cells

## Abstract

Hepatocyte spheroids are useful models for mimicking liver phenotypes *in vitro* because of their three-dimensionality. However, the lack of a biomaterial platform which allows the facile manipulation of spheroid cultures on a large scale severely limits their application in automated high-throughput drug safety testing. In addition, there is not yet a robust way of controlling spheroid size, homogeneity and integrity during extended culture. This work addresses these bottlenecks to the automation of hepatocyte spheroid culture by tethering 3D hepatocyte spheroids directly onto surface-modified polystyrene (PS) multi-well plates. However, polystyrene surfaces are inert toward functionalization, and this makes the uniform conjugation of bioactive ligands very challenging. Surface modification of polystyrene well plates is achieved herein using a three-step sequence, resulting in a homogeneous distribution of bioactive RGD and galactose ligands required for spheroid tethering and formation. Importantly, treatment of polystyrene tethered spheroids with vehicle and paradigm hepatotoxicant (chlorpromazine) treatment using an automated liquid handling platform shows low signal deviation, intact 3D spheroidal morphology and Z’ values above 0.5, and hence confirming their amenability to high-throughput automation. Functional analyses performance (i.e. urea and albumin production, cytochrome P450 activity and induction studies) of the polystyrene tethered spheroids reveal significant improvements over hepatocytes cultured as collagen monolayers. This is the first demonstration of automated hepatotoxicant treatment on functional 3D hepatocyte spheroids tethered directly on polystyrene multi-well plates, and will serve as an important advancement in the application of 3D tethered spheroid models to high throughput drug screening.

## Introduction

Primary hepatocytes have been widely used in applications ranging from therapeutics to drug safety testing studies. Hepatocytes used for these applications are often cultured on conventional monolayer systems although the cells remain viable for only short time periods and rapidly deviate from their differentiated phenotype under *in vitro* conditions^1–3^. Efforts to address challenges related to monolayer cultures have been aimed at developing 3D hepatocyte models ranging from spheroids formed in suspension^4^ to moderately adherent natural^5^ or artificially synthetic matrices^6–8^. In addition, micropatterned platforms, polyurethane foams and polymeric scaffolds have been used to form 3D spheroids^9–16^. Limited effort has been made to maintain and control the size of the spheroids in extended culture. This should be taken into consideration since collision of smaller spheroids to form larger ones would result in reduced or variable oxygen, nutrients, metabolites and drug access and eventually lead to necrosis in the inner core of the spheroid^17^. Additional challenges might involve difficulty in controlling variability in spheroid sizes and in manipulating floating spheroids for drug testing studies, especially if the system is to be used as automation based multi-well drug testing platform. Due to these challenges, spheroid cultures, despite their potential advantages, have not been used on a large scale and in a high-throughput manner for drug testing.

We have previously reported the formation of a 3D hepatocyte monolayer on membranes conjugated with Arg-Gly-Asp (RGD) and galactose in a 1:1 hybrid ratio^18^. This platform was further modified by carefully controlling the ratio of RGD and galactose ligands such that 3D hepatocyte tethered spheroids (TS) with uncompromised functions could be formed^19^. The tethered spheroids showed improved attachment and functional performance, which was comparable or better than conventional 2D cultures. Both the 3D monolayer and tethered spheroids platforms were developed on polyethylene terephthalate (PET) films.

Although PET films can act as a suitable substrate for ligand conjugation and hepatocyte culture, these films have to be handled with utmost care to avoid mechanical disruptions which could either dislodge or damage the 3D spheroids^20^, leading to inaccuracies in drug screenings. The level of care required to handle the PET tethered spheroids at a large scale and in an automated high throughput fashion necessitates costly and specialized robotic equipment. In order to enable high throughput drug safety screening on tethered spheroids at a low cost, we rationalized the direct tethering of hepatocyte spheroids onto the polystyrene multi-well plates to be the most accessible solution. From a chemical perspective, the activation of either the aliphatic backbone or phenyl rings on polystyrene for downstream chemical functionalization necessitates catalytic or high-energy approaches for the C-H bond cleavage^21–23^. Commercially, polystyrene well plates are functionalized by high energy ionizing techniques such as plasma treatment, corona discharge or UV ozone treatment to afford a 20–30% anionic oxygen or cationic nitrogen content on the polystyrene^24^. This level of functionality may be sufficient to address the general needs for a monolayer culture. However, as we have previously reported, the formation of tethered hepatocyte spheroids requires a delicate balance of specific receptor-associated ligands to afford optimal anchorage whilst maintaining cell aggregation^25^. Plasma or UV-ozone treatment results in non-specific increase in oxygen or nitrogen content which is difficult for use in specific chemical conjugation with biomolecules such as RGD and galactose ligands. In fact, the only reported instance of using non-specific hydroxyl groups for covalent bioconjugation requires an additional silanization step^26^. Unfortunately, this approach cannot ensure a homogeneous bioconjugation. Moreover, the equipment required for these treatments are not easily accessible.

Therefore, we leveraged our understanding of peroxide-mediated activation^27^ and our expertise in the functionalization of PET membranes, and successfully yielded reactive peroxide groups on polystyrene surfaces using only UV treatment in atmospheric conditions. Peroxide groups are known to rapidly decompose into radicals in the presence of light or heat^28–30^. In the current work, however, we show that these transient and unstable groups on polystyrene can be timely captured using a robust radical acceptor such as acrylic acid to achieve homogeneous poly (acrylic acid) handles for further functionalization of an otherwise inert substrate. A homogeneous poly (acrylic acid) polymeric coating on polystyrene substrate is the key to allowing a homogeneous covalent bioconjugation of RGD and galactose ligands onto polystyrene.

In addition to the functionalization of polystyrene multi-well substrates with RGD and galactose, the predominant focus of the existing work will be on the automation of tethered spheroid cultures for drug safety testing. Specifically, we are interested in investigating whether the polystyrene tethered spheroids display robust functionality, and whether they retain morphological integrity after being subjected to an automatic liquid handler system. The results of these studies may provide insights toward the design of an integrative automation system for drug screening on spheroid cultures.

## Materials and Methods

### Conjugation of galactose and/or RGD on PET film

Poly-acrylic acid (pAAc) was grafted onto the PET film (100 μm thickness; Sky Industrial Suppliers, Singapore) using a modified protocol^25,27^ for conjugating bioactive ligands. Briefly, a 10 cm PET film was cleaned in ethanol and air-dried before subjecting to argon plasma treatment (carried out in a SAMCO Basic Plasma Kit, SAMCO International Inc. operating at a radio frequency of 13.6 MHz). Argon (flow rate of 50 ml/min) was introduced into the chamber in the SAMCO kit. Chamber pressure maintained at 20 Pa. Electric power of 40 W for 1 min was used to generate plasma. The plasma-treated PET film was fully immersed in an aqueous solution of AAc (purged thoroughly with argon to remove oxygen) in a square petri dish, which was placed in a quartz tube. The quartz tube was subjected to UV irradiation for 30 min using a 400 W flood lamp in UV-F 400 unit (Panacol-Elosol GmbH, Steinbach, Germany) in a water bath at 28 °C. Residual homopolymer absorbed on the surface was removed by extensive washing of the PET film with deionized water. PET-pAAc film was cut into circular disks with diameter of 5 mm to fit into the 96-well plates. The films were conjugated with galactose and/or RGD using 1-ethyl-3-(3- dimethylaminopropyl) carbodiimide (EDC) and N-Hydroxysuccinimide (NHS) crosslinking. 0.25 mg AHG or 0.05 mg RGD in 75 ul phosphate buffer (0.1 M, pH 7.4) was added into each well for pure galactose or pure RGD conjugation respectively. 0.25 mg AHG and 0.025 mg RGD was added into each well to achieve RGD to galactose hybrid ratio of 1:2000. Following conjugation, the films were sterilized by soaking in 70% ethanol. Excess ethanol was removed by thorough rinsing with phosphate buffered saline (PBS, pH 7.4) before using for cell culture. The modified surface of the film was marked and manually transferred onto multi-well plates. Care was taken to ensure that the modified surface was facing the user.

### Conjugation of galactose and/or RGD on PS plate

The PS 96- well plate surface was pre-treated with UV for 10 minutes using a 400 W flood lamp in a UV-F 400 unit after which, AAc (purged thoroughly with argon) was added to each well. The reaction mixture was then subjected to UV irradiation to achieve UV- induced graft copolymerization of AAc with PS for 30 minutes. After the graft copolymerization, the plate was washed exhaustively with copious amount of deionized water to remove residual homopolymer absorbed on the surface. Subsequent steps for EDC and NHS crosslinking as well as galactose and/or RGD conjugation were similar to that used for the PET films as described in Section 2.1.

### Surface characterization of modified surfaces

The density of the grafted carboxylic groups on the PET films and PS wells was determined by a colorimetric method using Toluidine Blue O (TBO) staining as previously reported^27^. TBO staining was also used to determine homogeneity across wells and columns by measuring absorbance at 633 nm.

X-ray photoelectron spectroscopy (XPS) was used to qualitatively verify the pAAc grafting and ligand (galactose and/or RGD) conjugation onto PET and PS modified surfaces. VG ESCALAB Mk II spectrometer (VG Co., UK) with a MgKa X-ray source (1253.6 eV photons) at a constant retard ratio of 40 was used for measurements. Hydrocarbon peak at 285 eV were used as reference for the binding energies.

Static water contact angles of the pristine and modified PET and PS surface were measured using the sessile drop method on a goniometer (Contact Angle System OCA 30, Data Physics Instruments GmbH, Filderstadt, Germany) using the SCA20 software. Three sample readings from different surface locations were recorded and the average was used for analysis.

The surface morphologies of the pristine and modified PET films and PS surfaces were characterized by Atomic force microscope (AFM) with measurements taken at room temperature with a Dimension Icon AFM system with a Nanoscope V controller and Nanoscope analysis (Bruker, Santa Barbara, CA, USA). PeakForce QNM (Quantitative NanoMechanics) mode was used for nanomechanical measurements. This mode is based on the Derjaguin–Muller–Toropov (DMT) model on an AFM system under ambient conditions. Following a proper calibration procedure, ScanAsyst-air probe was used to scan the samples. The conditions were the following: nominal radius of 2 nm, nominal spring constant of 0.5 N/m, scan rate 1 Hz. The roughness profile determined by AFM was used to calculate the arithmetic mean of the surface roughness (Ra).

### Rat hepatocyte isolation and culture

Primary hepatocytes were harvested from male Wistar rats using a two-step *in situ* collagenase perfusion method according to protocols described previously^19,31^. Freshly isolated rat hepatocytes were seeded onto the different substrates at a density of 1 × 10^5^ cells/cm^2^ in 96-well microplates and cultured in 100 μl of William’s E culture medium supplemented with 1 mg/mL bovine serum albumin (BSA), 10 ng/mL epidermal growth factor, 0.5 mg/mL insulin, 5 nM dexamethasone, 50 ng/mL linoleic acid, 100 units/mL penicillin and 100 mg/mL streptomycin. Media was changed daily during the entire culture period. For collagen monolayer culture, neutralized 1.5 mg/ml Type I bovine collagen (Advanced BioMatrix, San Diego, CA, USA) was coated on the surface of the microplates.

### Primary human hepatocyte (PHHs) culture

Cryopreserved PHHs were purchased commercially from Life Technologies (Carlsbad, CA, USA) and BD Biosciences (Franklin Lakes, NJ, USA). Freshly thawed cells were cultured as described^32^ with daily culture media change. Hepatocytes from three different donors (three lots of commercial cells) were used for the experiments and all assays were performed after 72 hours of culture.

### Cell attachment and compatibility with automated system

Cell attachment and tethered spheroid formation was monitored using phase contrast microscopy for a period of seven days. In addition, CellTiter-Glo 3D Cell Viability Assay (Promega, Madison, Wisconsin, USA), which determines the number of viable cells present in a 3D cell culture/spheroid by quantifying the amount of ATP present, was used to test the variability in cell attachment across the plate and adaptation of the plate to an automated liquid handling system 5 days after cells seeding. Dispensing of compounds/reagents onto plates containing tethered spheroids was optimized using Agilent Bravo Automated Liquid Handling Platform. The following steps were optimized to ensure safe and accurate dispensing conditions- Aspirate volume, pre-aspirate volume, and the distance from the well bottom. Compatibility of the tethered spheroid plates to the automated liquid handling system was tested both with and without drug treatment: DMSO was used as negative control and Chlorpromazine (200 μM) as a positive control (paradigm hepatotoxicant). Following drug/DMSO treatment, the plates were incubated at 37 °C, 5% CO_2_ incubator for 24 hours. After that, CellTiter-Glo 3D was added to each of the wells and the relative light units (RLU) were quantified.

### Quantitative real-time PCR (qPCR)

RNA isolated using RNeasy Micro-kit (Qiagen, Hilden, Germany) was converted to cDNA using iScript cDNA synthesis kit (Bio-Rad Laboratories, Hercules, CA, USA). Real time PCR was performed using 7000 Fast Real-Time PCR System (Applied Biosystems, Foster City, CA, USA) and commercial primers (GeneCopoeia, Inc., Rockville, MD, USA). Glyceraldehyde-3-phosphate dehydrogenase (GAPDH) was used to normalize expression levels of all marker genes in order to account for differences in cell number.

### Albumin secretion and urea production

Human albumin enzyme-linked immunosorbent assay (ELISA) quantitation kit (Bethyl Laboratories, Inc., Montgomery, TX, USA) and Direct Urea Nitrogen Color Reagent and Direct Urea Nitrogen Acid Reagent (Standbio Laboratory, Boerne, TX, USA) were used to measure albumin and urea respectively according to manufacturer’s instructions and as described in^32^. Functional data was normalized to cell numbers quantified using the Quant-iT PicoGreen dsDNA Assay Kit (Life Technologies).

### Cytochrome P450 activity

Basal and induced activities as well as fold induction of three cytochrome P450 (CYP) enzymes, i.e. CYP1A2, CYP2B6 and CYP3A4 were determined by incubating PHHs with media containing inducers (40 μm β-naphthaflavone for CYP1A2, 1 mM phenobarbitol for CYP2B6 and 20 μm rifampicin for CYP3A4). After 48 hours of induction, medium was removed and the cells were incubated for 2 h at 37 °C with Krebs-Henseleitbicarbonate (KHB) buffer (118 mM NaCl, 1.2 mM MgSO_4_, 1.2 mM KH_2_PO_4_, 4.7 mM KCl, 26 mM NaHCO_3_, and 2.5 mM CaCl_2_) containing specific substrates (200 μM phenacetin for CYP1A2, 200 μM bupropion for CYP2B6 and 5 μM midazolam, for CYP3A4). The substrates and inducers were purchased from Sigma. The drug metabolite product was analyzed by Liquid Chromatography-Mass Spectrometry (MS: Finnigan LCQ Deca XP Max and Applied Biosystems 3200 QTrap, LC: Agilent 1100 series) according to procedures described in (Nugraha *et al*.^16^). CYP1A2 in rat hepatocytes were determined similarly with 40 μm β-naphthaflavone as the substrate. All basal and induced activities were normalized to cell numbers.

### Cell viability assay

Cell viability of hepatocytes was measured 24 hours after treatment with drugs using Alamar Blue cell viability assay according to manufacturer’s instructions. Drugs used for the cell viability assays (Acetaminophen, Troglitazone, Chlorpromazine and Diclofenac) were purchased from Sigma. Stock solutions of drugs were prepared in DMSO and then diluted in media to obtain desired working concentrations. Controls were treated with solvent alone (in absence of test compounds) and considered as 100% viability value.

### Inhibition of spheroid spreading

Hepatocytes were cultured as mentioned in section 2.1 and 2.2. Once spheroid formation was observed (2–3 days after cell seeding), cells were treated with DMSO or relevant hepatotoxicants (APAP/troglitazone) at different concentrations. 24 hour live cell imaging was performed starting 2 hours after drug addition using IN Cell Analyzer 2000 Imaging System (GE Healthcare Lifesciences, Buckinghamshire, U.K.). Images were acquired every 2 hours. Images acquired at 2 hours, 8 hours, 16 hours and 24 hours were used for further analysis. Individual spheroids were identified by image segmentation using Matlab Image Processing Toolbox and Statistical Toolbox (Mathworks) and the sizes of the spheroids were determined. Density plots were acquired in R (Version 3.1.2) to show the spheroid size distribution and percentage of spheroids within a certain diameter range at different drug concentrations was plotted in Excel.

### Measurement of spheroid size and distribution

Tethered spheroid images of both rat and human hepatocytes were taken 7 days after seeding. The number of tethered spheroids and spheroid diameter (μm) were calculated using ImageJ software. Only spheroids with a well-defined border were included in the calculations. Single cells, pre-spheroidal structures and spheroids near the edge of the images were ignored. For the determination of polydispersity coefficient, the spheroid diameters were fitted to a Gaussian curve on OriginPro and the constants derived from the Gaussian curve were used in the equation PD = w/2xc, where PD is polydispersity, w is full width of the Gaussian fit at 60% maximum and xc is the mean spheroid diameter. Since w/2 is the standard deviation of the Gaussian fit curve, the equation is essentially PD = standard deviation/mean size which is widely used for dispersity determination in nanocluster catalysis^33^. T-test of spheroid diameters of tethered spheroids was conducted using ANOVA on OriginPro.

### Statistical analysis

Data was collected from three technical replicates and three biological replicates. Paired two-tailed student t test was used for statistical comparisons. Results are expressed as mean ± standard error of mean (s.e.m).

### Ethical statement

All experimental methods and protocols using commercially purchased cryopreserved primary human hepatocytes (PHHs) was approved by the Institutional Review Board (IRB) in BMRC, A*STAR and performed following the Human Biomedical Research Act (HBRA). No informed consent was obtainable since these lots of primary human hepatocytes were commercial sources and human subjects were unidentifiable. Primary rat hepatocytes were isolated from animals maintained and experimented following NACLAR ethical guidelines and under the approval of the Institutional Animal Care and Use Committee (IACUC) in BMRC, A*STAR.

## Results

### PS multi-well plates are more amenable to UV-induced functionalization than PET films

**Figure 1:**
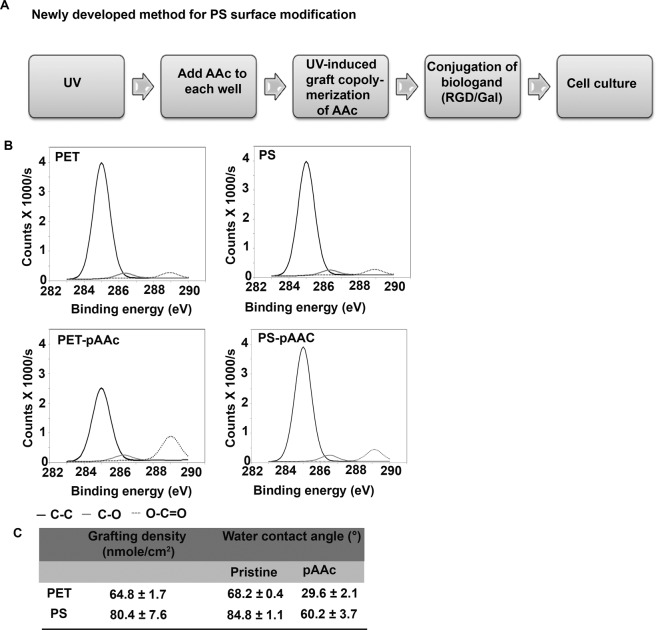
Characterization of successful polymer grafting on polystyrene surface. (**A**) Schematic diagram of the procedure for surface modification of polystyrene (PS) surface to enable UV-induced graft copolymerisation of acrylic acid (AAc) monomer using a two-step UV treatment method and consecutive ligand conjugation onto the polyacrylic acid (pAAc) grafted PS surface (PS-pAAc). (**B**) XPS analysis to verify grafting of pAAc on PS surface in comparison to previously used PET films (lower panel) compared to their respective pristine surfaces (upper panel) (**C**) Grafting density of pAAc on PS (in comparison to PET) determined by a colorimetric method using Toluidine Blue O (TBO) staining as described previously^27^ and water contact angle of PS-pAAc and PET-pAAc (in comparison to their respective pristine surfaces) measured by sessile drop method on a goniometer.

Automated multi-well large scale drug testing is challenging with our previous tethered spheroid model using PET substrates since these films are prone to mechanical perturbations which may affect spheroid integrity. Therefore, we decided to take the approach of tethering the spheroids directly onto the tissue culture substrate. However, polystyrene multi-well plates are widely known to be chemically inert and a homogeneous functionalization of polystyrene is challenging^26,34^. In order to achieve this, we modified the surface of PS well plate directly using a three-step UV treatment (Fig. 1A) and compared surface characterization (using quantitative and qualitative methods) to ensure that the modification on the PS well-plate is comparable to that on the PET film.

First, TBO dye was used to assess the successful grafting of pAAc on PS, based on the formation of ionic complexes between the –COOH groups of the grafted pAAc chains and the cationic dye. Our results showed uniform TBO staining throughout the well as well as across different wells (Supplementary Fig. 1), indicating that the surface grafting was uniform across different wells in the plate. Noteworthily, the grafting density of pAAc was 15.6 nmol/cm^2^ higher on PS than PET substrates (Fig. 1). This suggests that PS substrates may be more amenable to UV-induced acrylic acid grafting than PET substrates. This is unprecedented, considering that PET films consist of more reactive functional groups (i.e. polyesters) in comparison to PS, and are generally regarded as more amenable to functionalization than PS^35^.

Next, XPS was performed to qualitatively verify the grafting of pAAc on PS and compared to PET. The spectrum of PET-pAAc and PS-pAAc showed the same peaks for the pristine substrates (Fig. 1B, upper panel) and the relative intensity ratio of oxygen to carbon peaks was higher in PET-pAAc and PS-pAAc than their corresponding pristine surfaces (Fig. 1B, lower panel). The grafting density of –COOH on PS-pAAc was found to be 80.4 ± 7.6 nM/cm^2^ while the grafting density of –COOH on PET-pAAc was 64.8 ± 1.7 nM/cm^2^ (Fig. 1C, left-hand column). This indicates that the –COOH incorporation on the PS surface was successfully achieved.

Thirdly, water contact angle was measured to determine the change in surface hydrophobicity after pAAc grafting. Since the modification involves the incorporation of -COOH functionality which is known to be hydrophilic, incorporation of -COOH should lead to a decrease in water contact angle. Indeed, our results showed that the water contact angle for the pristine PS surface decreased from about 85° to 60° after the pAAc grafting, while the water contact angle for the pristine PET surface decreased from about 68° to 30° (Fig. 1C, right hand column). The data from the TBO staining, XPS analysis and water contact angle measurements confirms the successful grafting of pAAc on PS via the two-step UV treatment.

Finally, AFM was performed to investigate the topography of the modified surfaces both after pAAc grafting and conjugation of RGD and galactose bioligands. The pristine surface of both PET and PS was relatively smooth with evenly distributed small elevations (data not shown). Surface roughness is expected to increase compared to its pristine form due to chemical/radiation treatment. It has been shown that pAAc grafting increases surface stiffness (Fasce *et al*., 2008). Indeed, our results showed that the grafting of pAAc caused the average surface roughness of both pristine PET and PS surface to increase from 1.46 nm to 5.49 nm and from 2.89 nm to 11.3 nm respectively (Table 1). The conjugation of bioligands (galactose/RGD/galactose: RGD hybrid) on PS-pAAc surface further increased the average surface roughness, which was contrary to that of bioligand conjugated PET-pAAc surface. This difference in changes of surface roughness between bioligand conjugated PS-pAAc and PET-pAAc might be due to the overall surface property differences between PET and PS. The stiffness increased significantly after the grafting of pAAc on both PET and PS pristine surface and this shows that most of the surface is covered with –COOH (Table 1). The conjugation of the bioligands caused a decrease in the stiffness (with the exception of RGD). Interestingly, the modifications resulted in a greater change in both stiffness and surface roughness on the PS substrates compared to PET substrates. Specifically, PS stiffness decreased by 239 MPa and PS roughness increased by 28.51 nm whereas PET stiffness decreased by only 7 MPa and PET roughness increased by 1.07 nm. Taken together, the AFM results suggest that the bioligand conjugation was successful, and confirmed that the PS surfaces are more amenable to modification than PET films despite the use of a protocol originally developed for PET.

**Table 1:** Stiffness and roughness of unmodified (pristine) and modified polyethylene terephthalate (PET) and polystyrene (PS) surfaces after polyacrylic acid grafting (pAAc) and bioligand conjugation (Gal/RGD/Gal: RGD Hybrid) determined by AFM analysis.

	Stiffness (MPa)		Mean Root Square Roughness (Ra)/nm	
	PET	PS	PET	PS
Pristine	646	750	1.46	2.89
pAAc	970	901	5.49	11.3
Gal	728	600	1.48	15.3
RGD	734	967	2.90	25.7
Gal: RGD Hybrid	639	511	2.53	31.4

Gal: galactose, RGD: Arg-Gly-Asp.

### Tethered spheroids of low polydispersity can be formed using primary rat hepatocytes

In order to find out whether the functionalized polystyrene platform is able to support the formation of spheroids with uniform size and frequency, we obtained images across multiple wells of rat hepatocytes. Images and calculations from three representative wells are shown in Supplementary Fig. 2A and Table 2. Primary rat hepatocytes formed uniform spheroids by day 5. Images were taken at day 7. Spheroid diameter sizes were around 125.47 ± 1.32 μm (Table 2, Supplementary Fig. 2A). The average spheroid numbers in each well were 105 ± 12 (Table 2). On average, there were 68 ± 7 spheroids (more than 60% of total spheroids) with diameters lower than 150 μm. This is important since oxygen diffusion limits are around 150 μm and spheroids larger than 150 μm display necrotic cores and diminished function^36,37^.

**Table 2:** Summary of spheroid number, mean spheroid diameters and diameter dispersity calculated across three representative wells of tethered rat hepatocytes.

Rat Hepatocytes			
	Total Spheroid Number	Number of Spheroids < 150 μm	Mean Diameter (μm)
Donor A	101	69	126.00
Donor B	96	61	126.43
Donor C	119	74	123.96
Mean	**105**	**68**	**125.47**
Standard Deviation	**12**	**7**	**1.32**
	**Donor A**	**Donor B**	**Donor C**
Full width at 60% maximum of Gaussian curve (w)	31.18	77.96	50.77
Standard Deviation (w/2)	15.59	38.98	25.38
Mean Diameter (xc)	126.00	126.43	123.96
Polydispersity Coefficient (PD)	**0.12**	**0.31**	**0.20**

According to conventional definitions concerning dispersity of nanoparticles, systems displaying dispersity coefficients of less than 0.15 can be considered nearly monodispersed^33,38–40^. In our system, polydispersity calculations revealed that the spheroids were formed with polydispersity coefficients between 0.12 to 0.31 (Table 2). This suggests that tethered spheroids system is one of low polydispersity and, in some instances, close to a nearly monodispersed system. Employing the aforementioned conventions to our findings in Supplementary Fig. 2B, 90% of the size distribution of the tethered spheroids was within 20%, 51% and 33% of the mean size in Donors A, B and C respectively^33^. Taken together, these results suggest a good degree of control over the spheroid sizes and numbers across multiple batches.

### Polystyrene tethered spheroids are amenable to vehicle and paradigm hepatotoxicant treatment on an automated liquid handling platform

**Figure 2:**
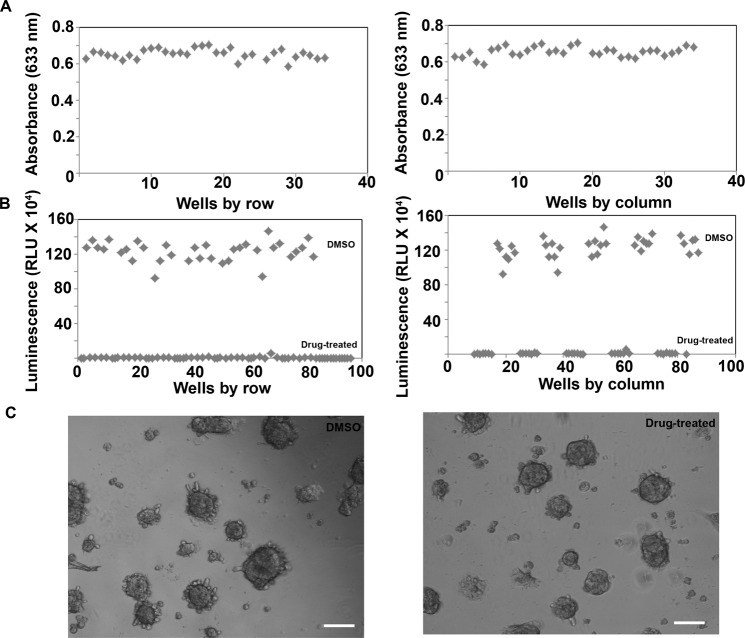
Adaptability of modified PS plates for automated platforms (**A**) Quantification of TBO staining across different wells and columns using absorbance measurement at 633 nm. (**B**) Homogeneity of cell attachment and viability across wells and columns with and without drug treatment determined using CellTiter-Glo 3D Cell Viability Assay five days after cell seeding. (**C**) Phase contrast images showing intact spheroids tethered to the plate after performing media change and dispensing the compounds using the Automated Liquid Handling Platform. Scale bar: 200 μm.

In order for the tethered spheroid model to be used for high throughput screening, it is important that the modified plates are adaptable to an automated handling system. This includes ensuring that the surface conjugation is homogenous across different wells of the plate and the dispensing of the compounds or reagents using the automated system do not compromise the integrity of the spheroids. In order to test this, the TBO staining discussed in Section 3.1 was analyzed quantitatively to compare conjugation across wells in the plate. Our results showed that the absorbance values in wells both across rows and columns showed very little variation (Fig. 2A). The average absorbance was 0.65 ± 0.02 and the standard deviation was only 4.28% of the average. This suggests that the conjugation was homogenous, and could serve as a good platform for homogenous spheroid formation. Next, we seeded rat hepatocytes on these plates and measured cell viability using CellTiter-Glo 3D Cell Viability Assay upon treatment with 200 μM Chlorpromazine and vehicle control DMSO. Our results showed that DMSO treated wells showed higher signal (12.32 × 10^6^ RLU) in DMSO-treated wells both across rows and across columns while the drug treated wells showed a lower signal (1.55 × 10^4^ RLU) (Fig. 2B). Importantly, the variation in signal between different DMSO-treated wells and between different drug-treated wells were minimal (signal standard deviation: 9.23%). This shows that similar number of cells were attached in the form of spheroids in different wells along the rows and columns of a multi well plate and very little variability was observed from one well to another as long as treatment conditions remain unchanged. The minimal variation in readout across different wells is critical for a high-throughput system. Z’ values were above 0.5 which suggests that the assay could indeed be used in high-throughput screening (HTS) of compounds to identify hepatotoxicants.

A major technical challenge of conventional spheroids is media change due to the susceptibility of loose spheroids to motion. In order to ensure that the tethered spheroids were still anchored to the plate surface after media change, we imaged the plates after media was changed and cells were treated with fresh media containing drugs using the automated liquid handling system. Intact spheroids tightly anchored to the plate confirmed that the spheroids were not damaged or did not come loose as a result of media change or automated handling (Fig. 2C).

### Maintenance of functional performance of rat hepatocytes in polystyrene tethered spheroids

**Figure 3:**
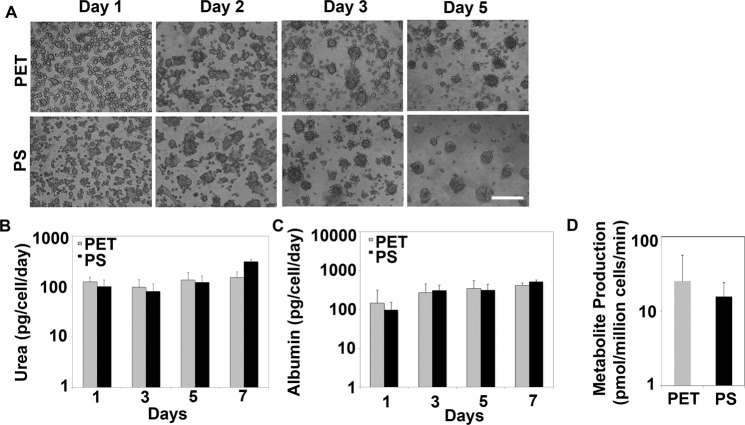
Characterization of primary rat hepatocytes on modified PS plates in comparison to previously used PET films. (**A**) Phase contrast images showing spheroid formation by primary rat hepatocytes on PET and PS surfaces. Scale bar: 200 μm (**B**–**D**) Urea, albumin and CYP1A2 basal activity (determined by metabolite production upon metabolism of phenacetin) respectively in rat hepatocytes cultured on PET and PS surfaces for seven days. Error bars represent s.e.m, n = 3 (3 biological replicates; 3 technical replicates).

Our previous results showed that tethered spheroids on PET film had better albumin production and comparable urea production compared to collagen sandwich after 5 days of culture^19^. We wanted to test if tethered spheroids on the PS system exhibited similar functional properties. Rat hepatocytes were seeded onto bioligand conjugated PET films and PS plates, spheroid formation was monitored and media was collected for functional assays at different time points for a week. Cells were also treated with CYP1A2 specific substrate to measure CYP1A2 basal activity. Our results showed that spheroid formation was observed within 3 days of cell seeding for both PET films and PS plates and could be maintained for at least five days (Fig. 3A). The dynamics and density of spheroid formation in the PS system was similar to that of the PET. Both albumin and urea production after 1, 3 and 5 days of culture were similar when tethered spheroids on PET and PS were compared. On Day 7, the urea production in PS was better (309.55 ± 35.13 pg/cell/day) than that on PET film (149.60 ± 47.40 pg/cell/day) (Fig. 3B) Similar trend was observed for albumin production (PS: 505.49 ± 67.04 vs PET: 413.80 ± 66.22 pg/cell/day) (Fig. 3C). No significant difference was observed in the CYP1A2 activity of hepatocyte tethered spheroids formed on PET films and PS plates (Fig. 3D). Overall, these results suggest that the adaptation of the tethered spheroid model from the PET to the PS system did not compromise the functional performance of the hepatocytes and similar or better functions could be maintained in the tethered spheroid model on the PS system.

### Maintenance of functional performance of primary human hepatocytes in polystyrene tethered spheroids

Once we confirmed the transfer of the conjugation system through material characterization and maintenance of functional performance in the polystyrene tethered spheroid system using rat hepatocytes, we analyzed if similar trends could be observed with primary human hepatocytes (PHHs). Average spheroid diameter of PHHs spheroids was 118.36 ± 15.38 μm and average spheroid number per well was 66 ± 4 (Supplementary Fig. 2A, Table 3). Polydispersity calculations reveal that the spheroids were formed with relatively low polydispersities (≤0.39). Statistical analyses revealed that the population diameter averages across all the wells of human and rat hepatocytes were not significantly different (Supplementary Fig. 2C).

**Table 3:** Summary of spheroid number, mean spheroid diameters and diameter dispersity calculated across multiple donors or batches of tethered human hepatocytes and compared to rat hepatocytes.

Human Hepatocytes						
	Total Spheroid Number		Number of Spheroids < 150 μm		Mean Diameter (μm)	
Donor A	63		40		133.57	
Donor B	69		49		102.82	
Donor C	65		52		118.69	
Mean	**66**		**47**		**118.36**	
Standard Deviation	**3**		**6**		**15.38**	
	**Rat Hepatocytes**			**Human Hepatocytes**		
Donor	**A**	**B**	**C**	**A**	**B**	**C**
Full width at 60% maximum of Gaussian curve (w)	31.18	77.96	50.77	102.90	45.42	53.68
Standard Deviation (w/2)	15.59	38.98	25.38	51.45	22.71	26.84
Mean Spheroid Size (xc)	126.00	126.43	123.96	133.57	102.82	118.69
Polydispersity (PD)	**0.12**	**0.31**	**0.20**	**0.39**	**0.22**	**0.23**

**Figure 4:**
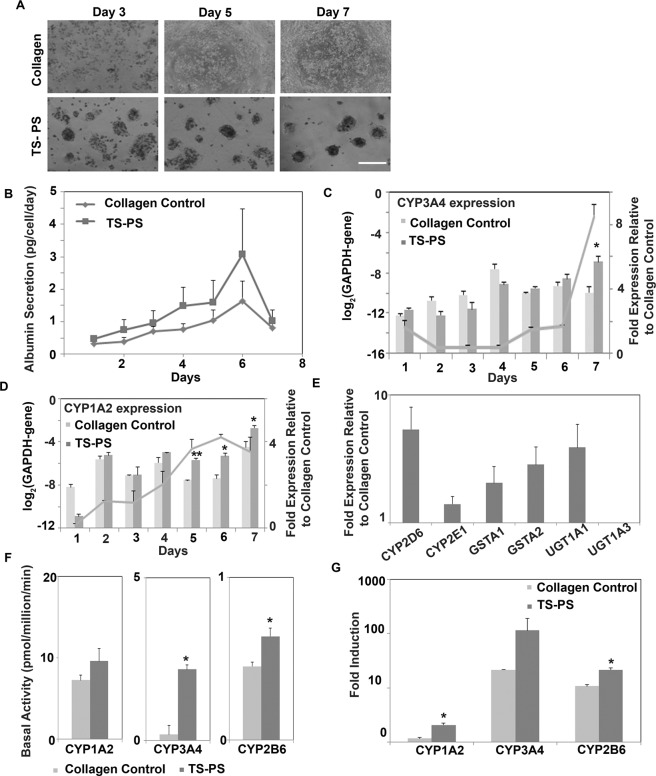
Characterization of primary human hepatocytes (PHHs) on modified PS plates. (**A**) Phase contrast images showing spheroid formation by primary human hepatocytes on modified PS plates. Spheroid formation was observed within five days, while hepatocytes cultured on collagen (2D control) remained as a flat sheet. Scale bar: 250 μm (**B**) Albumin production by PHHs when cultured on collagen substrate and as tethered spheroids on modified PS plates (TS-PS) over one week. Error bars represent s.e.m, n = 3 (3 biological replicates from Donors A, B and C; 3 technical replicates were used for each donor). (**C**,**D**) Respective gene expression of CYP3A4 and CYP1A2 in PHHs (Donor A). Data are represented relative to GAPDH expression. Grey line indicates fold expression in TS-PS compared to collagen control. *p < 0.05; **p < 0.01 (**E**) Relative gene expression of Phase I and Phase II enzymes in PHHs in TS-PS compared to collagen control (Donor A). (**F**) Day 5 Basal activity of CYP1A2, CYP3A4 and CYP2B6 in PHHs in TS-PS compared to collagen control (Donor A). (**G**) Induction of CYP1A2, CYP3A4 and CYP2B6 in PHHs upon 48 hours treatment of CYP specific inducers (Donor A). Data are represented as fold change in induced activity compared to the basal activity. (**C**–**G**): Error bars represent s.e.m, n = 3 (3 technical replicates from Donor A). Data from Donor B and C are presented separately in Supplementary Figs. 3 and 4 due to lot-to-lot variability in PHHs CYP activity and induction.

Next, functional performance of PHHs in the polystyrene tethered spheroid system was assessed and the results were compared with collagen control. Similar to the analysis of functional performance of tethered spheroids formed by rat hepatocytes, characterization of albumin secretion over a seven day period was carried out for PHHs tethered spheroids on PS substrates. PHHs exhibited similar spheroid formation as observed with rat hepatocytes (Fig. 4A). Spheroids formed within 3 to 5 days and could be maintained for at least up to a week. The albumin production in collagen culture and the polystyrene tethered spheroid (TS-PS) system was similar (Fig. 4B). In both cases, the albumin production increased gradually up to day 6. The tethered spheroids had a higher level of albumin production than the collagen control at day 6 (collagen control: 1.63 ± 0.62 pg/cell/day; TS-PS: 3.07 ± 1.41 pg/cell/day).

We analyzed the expression of CYP1A2 and CYP3A4 in polystyrene tethered spheroids and compared it to the collagen control over seven days. Due to variability in the gene expression and activity of CYPs in different lots of PHHs, data from different donors were analyzed separately. Tethered spheroids formed by PHHs from Donor A expressed either similar or higher levels of CYP1A2 and CYP3A4 compared to the collagen control (Fig. 4C,D). This was particularly prominent after the first 4 days of culture. CYP3A4 expression in TS-PS was 1.6 fold higher than collagen controls on days 5 and 6 and 8.5 fold higher on day 7 (Fig. 4C). CYP1A2 expression in TS-PS was 3.5–4.2 folds higher than collagen controls on days 5–7 (Fig. 4D). Similar trends were observed when expression of CYP1A2 and CYP3A4 in PHHs from Donor B was analyzed (Supplementary Fig. 3B,C). CYP3A4 expression in TS-PS was 2 folds higher than collagen control on days 6 and 7. CYP1A2 expression in TS – PS was 2.8–13 folds higher than collagen control on days 5–7.

Apart from CYP3A4 and CYP1A2, other CYPs such as CYP2D6 and CYP2E1 are also responsible for the Phase I metabolism of a wide variety of drugs. In addition, Phase II reactions, which involve conjugation with an endogenous substance (eg, glucuronic acid, sulfate, glycine) are also critical for the production of metabolites that are more polar and thus more readily excreted by the kidney (in urine) and the liver (in bile). Therefore, in addition to CYP3A4 and CYP1A2, we characterized the expression of CYP2D6 and CYP2E1 and the Phase II metabolic enzymes, Glutathione S-transferase (GST) and UDP glucuronosyltransferases (UGT) in the polystyrene tethered spheroids at day 6 and compared it to expression levels of the collagen control. With the exception of UGT1A3, the expression level of all other enzymes was 2–5.4 fold higher in TS-PS (Donor A) compared to the collagen control (Fig. 4E). Similarly, expression level of enzymes other than UGT1A3 was 1.2–2.7 fold higher in tethered spheroids formed by PHHs from Donors B and C (Supplementary Fig. 3D and Supplementary Fig. 4B).

In addition to CYP1A2 and CYP3A4 being critical isoforms among the CYP family for drug metabolism, CYP2B6 is also responsible for the metabolism of 4% of the top 200 drugs, and more substrates this enzyme are being discovered^41^. In order to ensure that the CYPs are not only expressed at the gene level, but also at the functional level, we investigated the basal activity of CYP3A4, CYP1A2 and CYP2B6 in TS-PS. Metabolite production after treatment with the respective CYP specific substrate was measured using LC/MS. Our results showed that the basal activity of CYP1A2, CYP3A4 and CYP2B6 in PS-TS on Day 5 was higher than that of the collagen controls (CYP1A2: 9.5 ± 1.56 vs 7.22 ± 0.65; CYP3A4: 2.18 ± 0.13 vs 0.18 ± 0.28; CYP2B6: 0.64 ± 0.05 vs 0.45 ± 0.02 pmol/million cells/min) (Donor A, Fig. 4F). Similar increases in basal activity was observed in Donor B (Supplementary Fig. 3E). This increase in basal activity in CYP1A2 and CYP3A4 is consistent with the increase in fold expression at gene level as determined by qPCR analysis (Fig. 4C).

Apart from CYP activity, CYP induction is an important functional endpoint for assessing hepatocyte performance. Hence, we investigated the potential induction of CYP activity in the TS-PS system. Metabolite formation in untreated cells (basal activity) and in cells treated with prototypical inducers (induced activity) was measured by LC/MS. The fold change in metabolite production in induced vs non-induced samples is referred to as fold induction. Following exposure to the prototypical inducers, the fold induction levels of CYP3A4, CYP1A2 and CYP2B6 in PS-TS were 114.42 ± 25.42, 2.06 ± 0.18 and 21.38 ± 1.83 respectively (Fig. 4G), which were higher than the fold induction in the collagen control (21.52 ± 0.29, 1.17 ± 0.03 and 10.70 ± 0.61). Similar increases in fold induction was observed in Donors B and C (Supplementary Figs. 3F and 4D).

**Figure 5:**
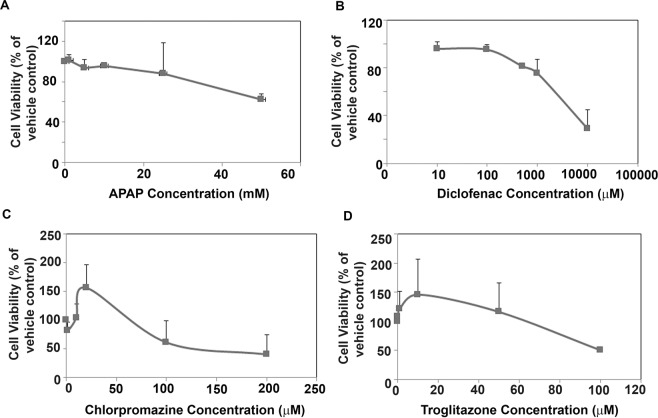
Treatment of TS-PS with four model hepatotoxicants. The cell viability of PHHs in TS- PS was assessed by Alamar Blue assay after exposure to different concentrations of Acetaminophen (**A**), Diclofenac (**B**), Chlorpromazine (**C**) and Troglitazone (**D**). Lines in each panel represent cell viability of PHHs at different concentrations of hepatotoxicants. Cell viability is expressed as a percentage of cells treated with solvent alone. Error bars represent s.e.m, n = 3 (3 biological replicates from Donors A, B and C; 3 technical replicates were used for each donor).

Finally, we treated the PHHs spheroids on the TS-PS system with four paradigm hepatotoxic compounds and assessed their viability at different drug concentrations (Fig. 5). The IC_50_ values of the four model drugs (Acetaminophen: 50 mM, Diclofenac: 4000 μM, Chlorpromazine: 122 μM and Troglitazone: 100 μM) derived from the dose-dependent testing correlate well with reported *in vitro* IC_50_ and *in vivo* LD_50_ values^42–45^.

In summary, these results suggest that the synthetic functions and expression of Phase I and Phase II drug metabolic enzymes, CYP3A4, CYP1A2 and CYP2B6 basal activity and induction in TS-PS system were higher than the collagen control and the TS-PS was able to successfully identify four paradigm hepatotoxicants (Fig. 5).

## Discussion

In this study, we have successfully functionalized inert polystyrene multi-well plates and conjugated an optimized ratio of RGD and galactose so as to tether hepatocyte spheroids directly onto polystyrene well plates. Additionally, we demonstrated the amenability of the polystyrene-tethered spheroid system to the automated testing of a paradigm hepatotoxicant Chlorpromazine. We have also assessed the functions of the tethered spheroids to be better than that of 2D monolayer collagen cultures, and comparable to spheroids formed on PET films.

Several comprehensive reviews aptly summarize the advantages and drawbacks of existing *in vitro* liver models for hepatotoxicity testing^46,47^. The gold standard, PHHs cultured in monolayer, is faced with issues related to loss of functions^3,48^. These challenges have partially been overcome by culturing hepatocytes in 3D models. However, these 3D models involve the use of hydrogels, scaffolds, and complex systems such as microfluidic devices. These models lack established standards and are not easily adjustable to high throughput screening^46,47^. Having a 3D liver model which can overcome drawbacks of 2D monolayer cultures and, at the same time, be adjusted for high throughput would allow 3D spheroid models to be used more extensively for drug safety testing.

In addition to answering the aforementioned problems, the tethered spheroid model developed in this study uses only synthetic polymeric materials and circumvents the use of exogeneous extracellular matrices such as collagen which is typically used in 2D collagen sandwich models. This reduces the overall variability of the system. Lot-to-lot variability is a prevalent problem in 2D and certain 3D culture systems which use components from natural sources. An example is collagen which is widely used in hepatocyte cultures^46,47^ but is prone to lot-to-lot variability^49^. Combining these systems with PHHs, which have their inherent donor variability, further increases the overall variability and negatively impacts the reliability of drug screening endeavors.

Existing spheroid systems encounter problems associated with insufficient cell yields and necrotic core due to uncontrolled aggregation. Spheroid models can either be single-spheroid based such as the commercially available systems developed by Corning and InSphero^50,51^ or multiple-spheroid based^3,52^. One potential limitation of a single spheroid system is the lack of sufficient cells for assays and usually requires combining several spheroids from several wells as illustrated in the application note from Corning^50^. Multiple spheroid based systems may overcome this limitation. However, multiple spheroid systems require the use of scaffolds or gels which would make adaptation to high throughput more challenging^47^. Although there are reports of multiple spheroids in a scaffold/gel-free system, these spheroids are likely to aggregate, eventually leading to the formation of larger spheroids with necrotic cores^52^. Necrotic cores in spheroids are due to poor diffusion of oxygen and important nutrients which have a diffusion limit of around 150–200 μm^36,37^.

In contrast to existing spheroid systems which have proven problematic, the polystyrene tethered spheroid system developed in this study overcomes these limitations through anchorage of multiple spheroids of relatively similar sizes to the surface of the multi-well plate. This allows the model to maintain multiple 3D hepatocyte spheroids in a scaffold/gel free high throughput adaptable system without the risk of spheroid aggregation.

Importantly, the system presented in this study allows for good control over spheroid sizes for both rat and human hepatocytes. In particular, tethered spheroids are formed with low diameter dispersities between 12% to 39%, and consistent diameters in the range of 118–125 μm (Tables 2, 3). By the conventional definition of dispersity in nanocluster analysis, the tethered spheroids achieved herein are of low polydispersity and, in some instances, close to a nearly monodispersed system^33^. The ability to constrain the spheroids to a diameter range below 150 μm consistently is important to the functional performance of the hepatocytes in three-dimensional culture since spheroids larger than 150 μm experience core necrosis, and diminished function attributable to poor core penetration of oxygen and important nutrients^36,37,53–55^.

In our previous studies to elucidate the mechanism of hepatocyte spheroid formation on PET membranes, we have identified 4 distinct stages leading to 3D spheroid formation namely, (1) small cell-aggregates, (2) ‘island-like’ clusters, (3) pre-spheroid monolayer and (4) 3D spheroids^18,25^. The duration at which the cells rearrange as pre-spheroid monolayer before transitioning to 3D spheroids is heavily dependent on the relative ratios of asialoglycoprotein (ASGPR)-binding galactose and integrin-binding RGD ligands. The ratios of galactose and RGD ligands modulate cytoskeletal rearrangement and phosphorylation of the focal adhesion kinase (FAK) proteins which are important features in cell migration and tethering processes. We believe that the spheroid size control in our present system may have been a culmination of these phenomena.

**Figure 6:**
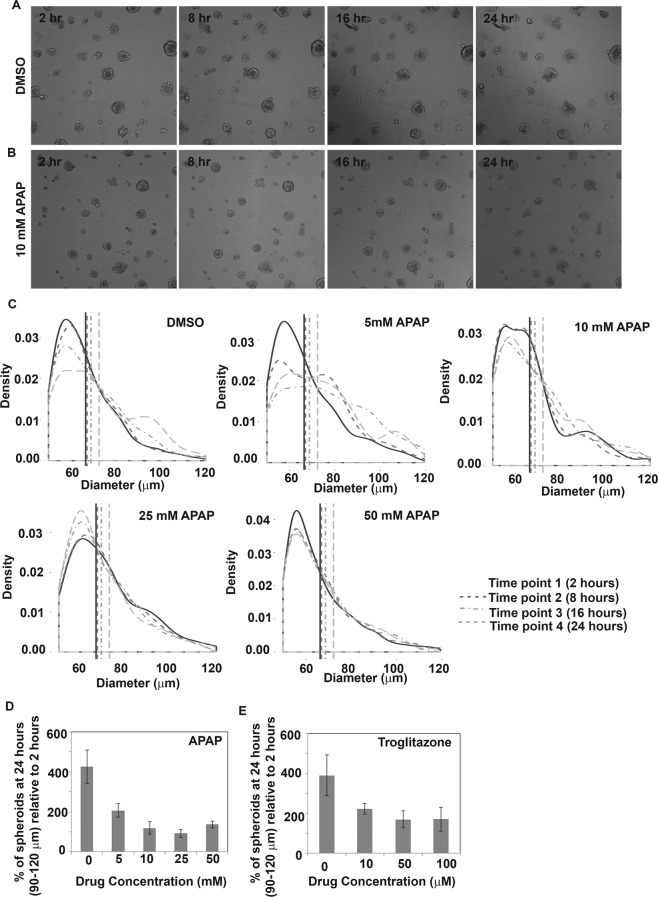
Live cell imaging demonstrating the inhibition of tethering process of spheroids upon treatment with hepatotoxicants. Phase contrast images showing tethered spheroid formation on modified PS plates without (**A**) and with addition of hepatotoxicant, (10 mM APAP) (**B**). In untreated controls (upper panel), blebs, a typical feature of cell adhesion and migration was observed as early 2 hours upon initiation of live imaging. This was not observed when spheroids were treated with APAP (lower panel). Scale bar: 250 μm (**C**) Diameters of the spheroids upon treatment with different concentrations of APAP and untreated control (DMSO) were quantified at 4 different time points (2 hours, 8 hours, 16 hours and 24 hours upon drug addition). Higher density of spheroids with diameters >80 μm are observed in DMSO treated samples at 24 hours. This is not observed when the spheroids are treated with APAP, especially at concentrations >5 mM. Spheroids of diameter 90–120 μm at 24 hours were measured and expressed as percentage relative to 2 hours upon treatment with different concentrations of APAP (**D**) and troglitazone (**E**). Error bars represent s.e.m, n = 3 (3 biological replicates; 3 technical replicates).

One interesting phenomenon that we observed during the course of this study was that perturbation of the tethering process of the spheroids using paradigm hepatotoxicants affected spheroid diameters and numbers and inhibited the tethering process (Fig. 6A). In untreated controls, blebs, a typical feature of cell adhesion and migration^56^, was observed around the spheroids indicating that the spheroids were attached to the substratum. In order to analyze this phenomenon further, the diameter of the spheroids at different time points were monitored over 24 hours upon treatment with DMSO (vehicle control, Fig. 6B) and different concentrations of Acetaminophen (APAP, Fig. 6C) through live imaging. We observed that hepatocytes treated with DMSO continued to reorganize and to form the tethered spheroids with typical blebbing associated with lamellipodia, stress fibers and focal adhesions. This resulted in a higher number of spheroids with diameters 80–100 μm (Fig. 6C) indicated by a shift in the curve at 24 hours (time point, T4). This did not occur when the hepatocytes were treated with >5 mM APAP and no difference in the surface area of the curve or diameter of spheroids was observed. When this phenomenon was quantified using different concentrations of APAP, we observed a concentration dependent decrease in the number of spheroids of diameter 90–120 μm (Fig. 6D), indicating the lack of cellular dynamic changes and reorganization upon drug treatment. Interestingly, spheroids of diameter 90–120 μm decreased by 50% compared to untreated control when hepatocytes were treated with 5 mM of APAP. If these results are correlated with typical methods for calculating IC_50_, i.e. concentration at which 50% of cells are viable, IC_50_ of APAP using changes in cellular dynamics would be 5 mM. This suggests that cellular dynamic changes might be a more sensitive marker for detecting hepatotoxicity compared to conventional cell viability assays which yield an IC_50_ of 8–40 mM^57,58^. Similar trend was observed with the paradigm hepatotoxicant troglitazone (Fig. 6E). Spheroids of diameter 90–120 μm decreased by 50% compared to untreated control when hepatocytes were treated with 10 μM of troglitazone. IC_50_ of troglitazone has been reported to be 140 μM^59^. Although this is a very interesting phenomenon, further studies with additional drugs need to be conducted to verify the sensitivity and specificity of using this method for hepatotoxicity detection.

The anchorage of the spheroids directly to the polystyrene multi-well plate allows the tethered spheroid system herein to be used in automated systems without affecting the quality of the spheroids. As previously mentioned, spheroids remain tethered even after automated drug treatment (Fig. 2C) and this suggests that the spheroids are stably immobilized to the plate surface. In some instances of gel/scaffold-free spheroid cultures, the loose spheroids are compromised in terms of its integrity as they are overly exposed to physical disruptions in an automated liquid handling system. They may even be completely washed off from the well plates. This is also applicable to commercially available single spheroid systems discussed above. These problems greatly affect the accuracy of the output in high throughput screenings if they are not circumvented.

Noteworthily, the functionalized polystyrene well plates described in this work are highly versatile and can support the formation of both rat and human hepatocyte spheroids. We compared the functional performance of rat hepatocytes in our system to that of the state of the art. CYP1A2 activity has been reported to be 0.1 nmol/min/mg protein which corresponds to 20 pmol/million cells/min based on 0.2 ng protein/cell^58^. The CYP1A2 is our study was comparable (25 and 16 pmol/million cells/min in PET films and PS plates respectively). Urea production has been reported to be 3–8 μg/μg DNA/24 hrs which corresponds to 18–48 pg/cell/day based on 6 pg DNA per cell^60^. In our system, the hepatocytes produced 99–300 pg/cell/day urea, which is significantly higher than reported values. Similarly, albumin production, which has been reported to be 0.3–2 μg/μg DNA/24 hrs (corresponding to 1.8–12 pg/cell/day)^60^ or 2.4–48 pg/cell/day^61^, was higher in our system (100–500 pg/cell/day throughout the 7 days).

There have been limited reports of human hepatocytes cultured in 3D systems. One of the few reports used biodegradable poly(l-lactic acid) (PLLA) polymer scaffolds and in a flow bioreactor to culture isolated human hepatocytes^62^. The albumin production was reported to be 2.64 pg/cell/day^62^. In our system, we observe similar level of albumin production (0.5–3 pg/cell/day), suggesting that the tethered spheroid model supports hepatocyte functionality without the need for perfusion or other complicated additions to the model.

CYP3A4 activity in PHHs has been reported to be 40–800 pmol/mg protein/min corresponding to 8–160 pmol/million cells/min based on 0.2 ng protein/cell^63,64^. This wide variation in activity levels can be attributed to donor variability and difference in culture conditions^63,64^. The CYP3A4 basal activity in our system was approximately 12.5 pmol/million cells/min which was within the range of reported values. PHHs from Donor A showed a lower CYP3A4 basal activity (2 pmol/million cells/min), likely due to be donor variability. Importantly, CYP3A4 induction has been reported to be 2–6 fold^63,64^ with only rare cases of induction above 10 fold^63^. Our system shows a much higher fold of CYP3A4 induction (114.4 fold) compared to reported values as well as the 2D collagen control. The basal activity and induction levels of CYP2B6 in the tethered spheroid model were similar to reported values as well as our internal 2D collagen control^63,64^. CYP1A2 activity in PHHs has been reported to be 0.73–4.15 pmol/mg protein/min corresponding to 0.15–0.83 pmol/million cells/min based on 0.2 ng protein/cell^63,64^. The CYP1A2 activity in the tethered spheroid model was 9.56 pmol/million cells/min, which is higher than reported values as well as the 2D collagen control.

## Conclusion

Hepatocyte spheroids tethered directly on a polystyrene multi-well plate were subjected to automated hepatotoxicant treatment with minimal disruption to their 3D morphological integrity and displayed high readout accuracy. In comparison to conventional 2D cultures, the functionalized polystyrene well plates supported the formation of functionally superior spheroids from both rat and human hepatocytes. Noteworthily, the polystyrene well plates were efficiently functionalized with a homogeneous layer of poly (acrylic acid) handles for bioconjugation despite the inert chemical properties of polystyrene. The facile UV-induced polystyrene modification, convenient spheroid formation, and morphological stability of the spheroids under automated pipetting systems are valuable features that may help to significantly advance the field of high throughput hepatotoxicity screening on 3D tethered spheroid cultures.

## Supplementary information

Supplementary information
