# Supplementary material for: Tethered primary hepatocyte spheroids on polystyrene multi-well plates for high-throughput drug safety testing
Source: Sci Rep. 2020 Mar 16;10:4768. doi: 10.1038/s41598-020-61699-4 (PMC7075904; doi:10.1038/s41598-020-61699-4)
Supplement: Supplementary file 1 — Supplementary information [file 41598_2020_61699_MOESM1_ESM.docx]

**SUPPLEMENTARY INFORMATION**

**Tethered primary hepatocyte spheroids on polystyrene multi-well plates for high-throughput drug safety testing.**

Farah Tasnim^a^, Nisha Hari Singh^a^_,_ Elijah Keng Foo Tan, Jiangwa Xing_,_ Huan Li, Sebastien Hissette, Sravanthy Manesh, Justina Fulwood, Kapish Gupta, Chan Way Ng_,_ Shuoyu Xu, Jeffrey Hill and Hanry Yu*

^a^ Co-first authors

* Corresponding author: Tel: +6568247103, Fax: +68749526; [hyu@ibn.a-star.edu.sg](mailto:hyu@ibn.a-star.edu.sg)


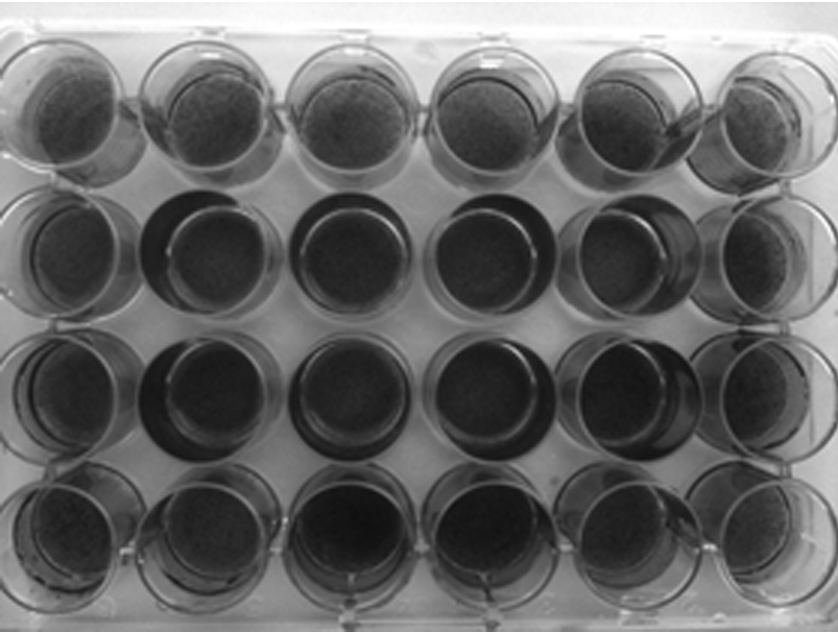
Supplementary Figure 1: Image of multi-well plates showing uniform staining with TBO


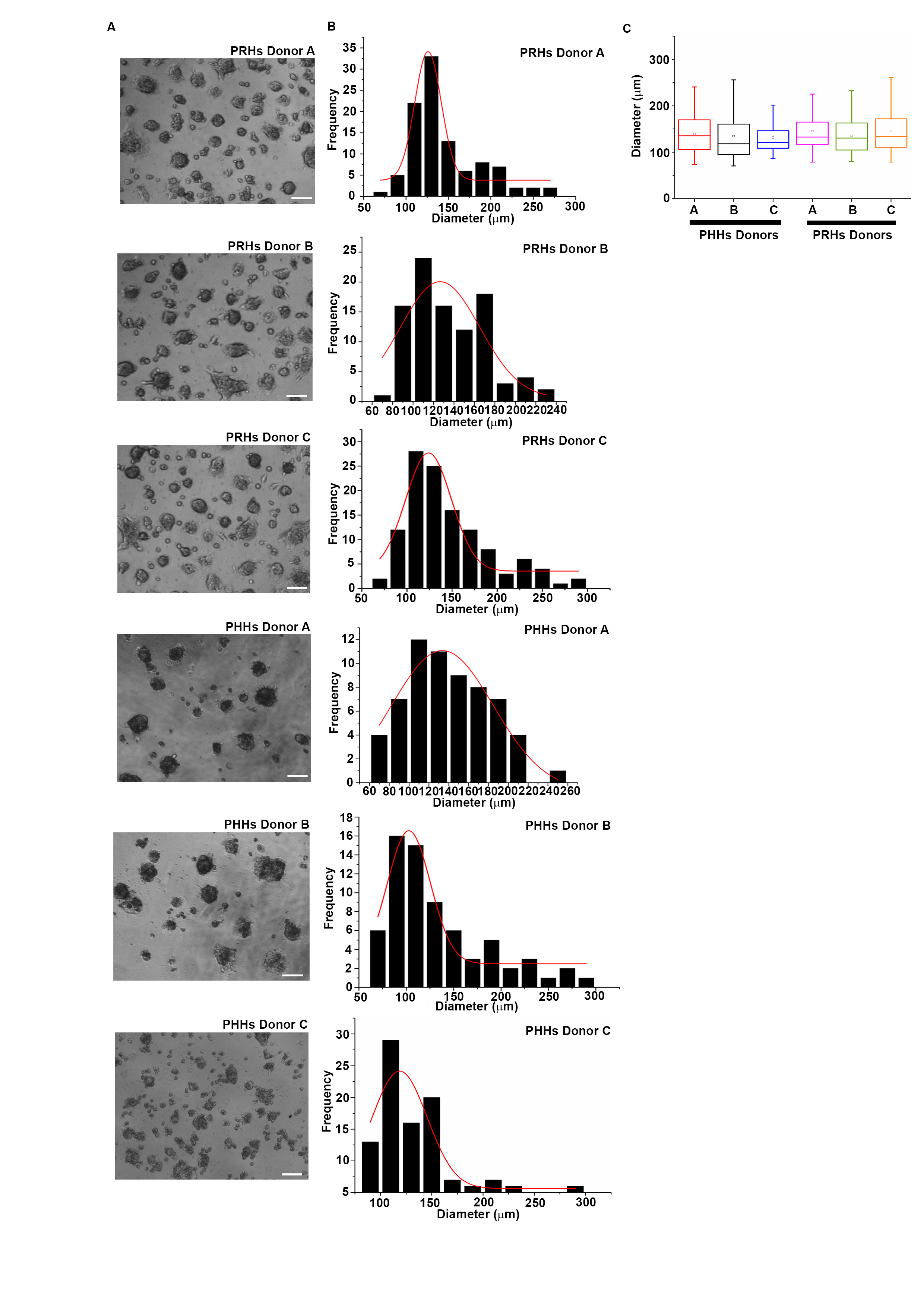


Supplementary Figure 2: (a) Phase contrast images showing rat and human hepatocyte tethered spheroids at Day 7. Scale bar: 200 μm (b) Histogram and Gaussian fits of the diameters of tethered spheroids across the sampled wells. (c) T-test of average diameter of tethered spheroids across all the wells and batches (both human and rat tethered spheroids) at the 0.05 level. There are no significant differences in the average diameters of both rat and human tethered spheroids across all the sampled wells. PHHs: primary human hepatocytes; PRHs: primary rat hepatocytes. Error bars represent s.e.m, n =3 (3 biological replicates; 3 technical replicates).





Supplementary Figure 3: (A) Phase contrast images showing morphology of PHHs from Donors A (left panel) and B (right panel). Images were taken 6 hours after seeding. Scale bar: 50 μm. (B, C) Respective gene expression of CYP3A4 and CYP1A2 in PHHs (Donor B). Data are represented relative to GAPDH expression. (D) Relative gene expression of Phase I and Phase II enzymes in PHHs in TS-PS compared to collagen control (Donor B). (E) Day 5 Basal activity of CYP1A2, CYP3A4 and CYP2B6 in PHHs in TS-PS compared to collagen control (Donor B). (F) Induction of CYP1A2, CYP3A4 and CYP2B6 in PHHs upon 48 hours treatment of CYP specific inducers (Donor B). Data are represented as fold change in induced activity compared to the basal activity. Error bars represent s.e.m, n =3 (3 biological replicates; 3 technical replicates). *: p<0.05; **: p<0.01


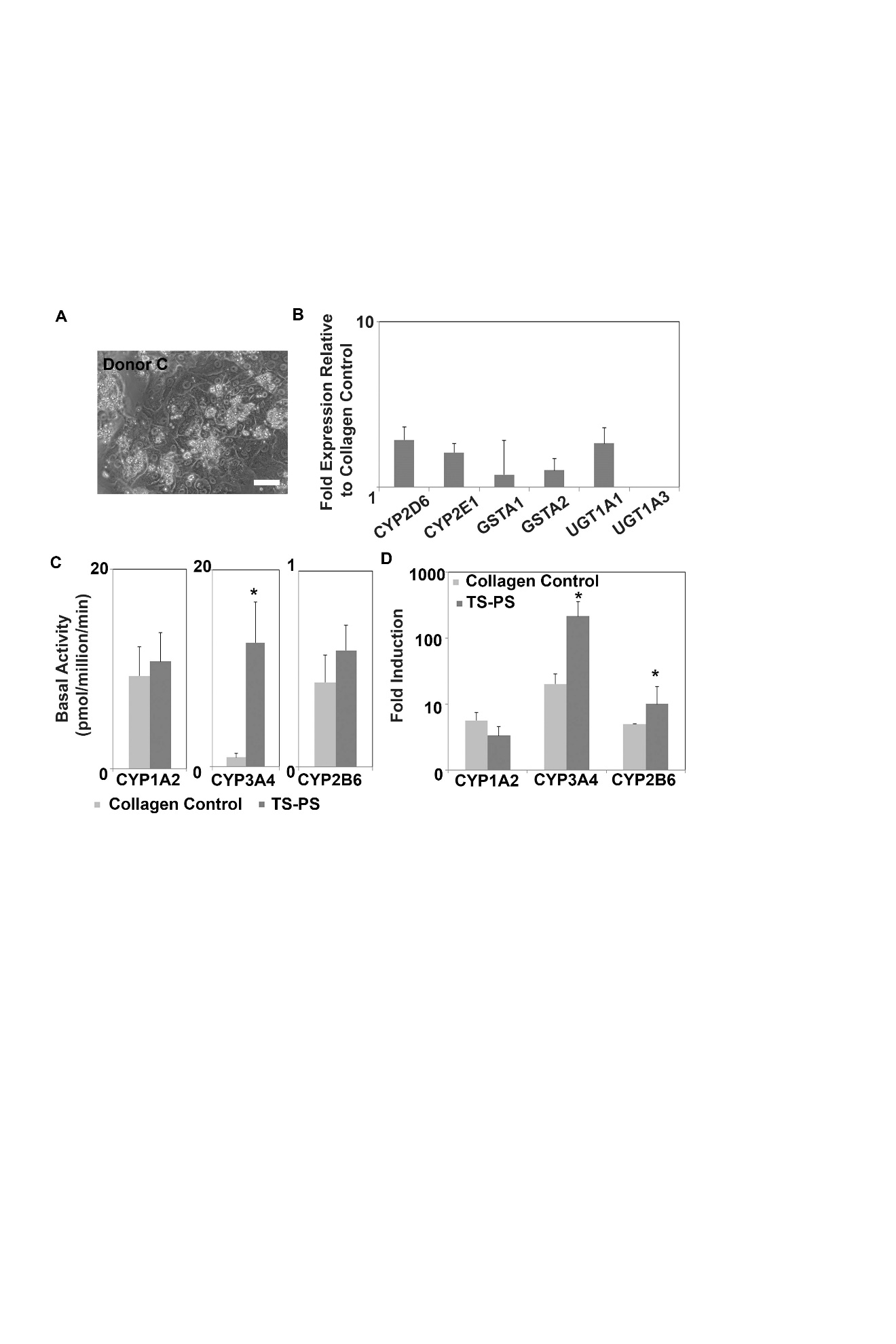


Supplementary Figure 4: (A) (Phase contrast images showing morphology of PHHs from Donors C. Images were taken 6 hours after seeding. Scale bar: 50 μm. (B) Relative gene expression of Phase I and Phase II enzymes in PHHs in TS-PS compared to collagen control (Donor C). (C) Day 5 Basal activity of CYP1A2, CYP3A4 and CYP2B6 in PHHs in TS-PS compared to collagen control (Donor C). (D) Induction of CYP1A2, CYP3A4 and CYP2B6 in PHHs upon 48 hours treatment of CYP specific inducers (Donor C). Data are represented as fold change in induced activity compared to the basal activity. Error bars represent s.e.m, n =3 (3 biological replicates; 3 technical replicates). *: p<0.05.
